# From lipofuscin accumulation to cellular dysfunction: a focus on liver pathophysiology

**DOI:** 10.1007/s00418-026-02502-9

**Published:** 2026-06-25

**Authors:** Filip Braet, Eddie Wisse, Ger H. Koek, Gerald J. Shami, Amy Li

**Affiliations:** 1 School of Medical Sciences (Molecular Biomedicine), The University of Sydney, Sydney, NSW Australia; 2 Australian Centre for Microscopy & Microanalysis, The University of Sydney, Sydney, NSW Australia; 3 Division of Nanoscopy, Multimodal Molecular Imaging Institute, University of Maastricht, Maastricht, The Netherlands; 4 School of Nutrition and Translational Research in Metabolism, Maastricht University, Maastricht, The Netherlands; 5 School of Medical Sciences, The University of Sydney, Sydney, NSW Australia; 6 Health Hub, Torrens University Australia, Sydney, NSW Australia; 7 Department of Rural Clinical Sciences, La Trobe Rural Health School, La Trobe University, Melbourne, Victoria Australia

**Keywords:** Aging pigment, Cellular stress, Chronic disease, Histochemical stains, Histopathological marker, Liver disorder, Primary and secondary lysosomes, Lipofuscin structure–function, Volume electron microscopy

## Abstract

In this review, we summarize the data on the cellular pigment lipofuscin that accumulates in liver tissue over time, due to aging and cellular stress. Despite the presence of these typical subcellular inclusions under various conditions, relatively little is known about their origins, roles, and effects on liver cell and tissue health. Pathologists use the presence of lipofuscin, in combination with other markers, to achieve differential diagnosis across various diseases. Routine histological stains reveal characteristic irregular shaped intracellular inclusions of lipofuscin that cannot be missed. Moreover, lipofuscin is autofluorescent and in transmission electron microscopy it appears in the cytoplasm as irregularly shaped structures containing fat and floccular material with varying electron density. Herein, we discuss the current state of knowledge concerning the origin and function of this pigment in the liver. Lipofuscin can distinctively be found in liver, although it has also been reported in cells in the heart, brain, and eye. Its biochemical composition is heterogeneous and varies depending on the tissue and the age of the organism. The liver parenchymal cells have efficient cellular waste disposal mechanisms, but they are still susceptible to aging. Lipofuscin accumulation in the liver may result from ongoing oxidative damage and impaired hepatic detoxification leading to cellular stress.

**Supplementary Information:**

The online version contains supplementary material available at 10.1007/s00418-026-02502-9.

## Introduction

Lipofuscin was first described in 1842 but the term was not coined until much later (Borst 1922; Hannover 1842; Hueck 1912). Lipofuscin derived its origin from ‘*lipo-*’ and ‘*-fuscus*’ meaning ‘fat that is dark, black/brown’. Throughout the twentieth century, the term lipofuscin was considered interchangeable with ceroid, ceroid lipofuscin, lipopigments, lipochromes, and more colloquially, the aging or wear-and-tear pigment (Nandy 1982). The discussion about these terms concerns the accumulation during aging, while ceroids refer to a pathophysiological process (Tohma et al. 2011; Seehafer and Pearce 2006). Pearse proposed that ceroids were simply lipofuscin in early-stage oxidation (Pearse 1985). Brunk and Terman reasoned that while the arbitrary distinction may be etiologically valid, the properties and mechanism of formation are comparable (Brunk and Terman 2002a).

**Fig. 1:**
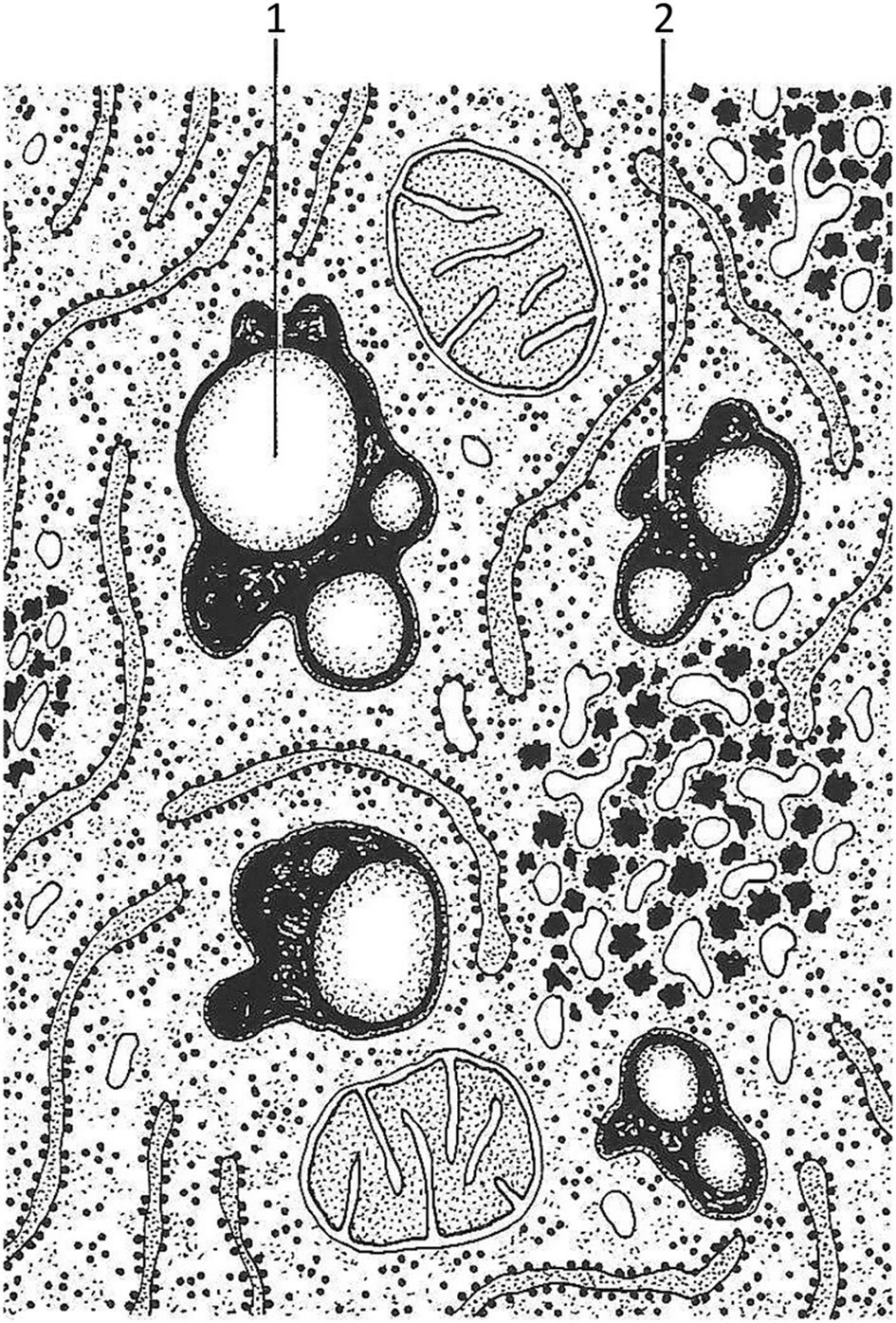
Drawing of the cytoplasm of liver parenchymal cells showing irregularly shaped, heterogeneous lipofuscin pigments scattered between the rough endoplasmic reticulum and mitochondria.* Legend*: 1, lipid droplet within lipofuscin pigment; 2, lipofuscin pigments contain also irregularly shaped particles and lamellae, probably representing remnants of digestive processes of former lysosomes. Note, illustration is drawn to scale, × 37,000. (Reprinted and modified with permission from Springer-Verlag (Krstić 1979): Copyright 1978–1979)

Lipofuscin has been extensively studied in neural and retinal organs given their direct involvement in age-related pathology such as macular degeneration, Alzheimer’s disease, and Parkinson’s disease (Nandy 1982). The role of lipofuscin in the liver seems to be more related to aging or stress than to disease. Recent evidence, however, infers the involvement of lipofuscin in liver disease (Saif et al. 2020). Cell biologists and pathologists have recognized the importance of the direct structural relationship of secondary lysosomes or residual bodies and excess lipids. From early on, the presence and buildup of lipofuscin have been regarded as a morphological indicator of organ adversity. Underpinning their importance, detailed descriptions were included in relevant atlas textbooks for students of medicine and biology, highlighting the significance of lipofuscin in cell function and organ fate. Figure 1 illustrates such a historical example of the ultrastructure of lipofuscin in liver tissue (Krstić 1979).

In this review, we will discuss the biology of lipofuscin, including the visualizations that characterize these pigment inclusions. Next, we will explore the presence of lipofuscin in liver parenchymal cells in disease conditions, aging, and oxidative stress. Nowadays, pathologists recognize lipofuscin accumulation within liver parenchymal cells as a clinical hallmark of overall liver adversity.

## Lipofuscin biology

Lipofuscin can be found throughout the cell interior and is present in a number of cell types as membrane-bound bodies, surrounded by a lysosomal membrane approximately 10 nm thick. They contain aggregates of electron-dense material ranging in size from 0.5 to 5 µm in diameter. These inclusions seem to be permanent, nondegradable, nor are they ejected from the cell cytoplasm. They have a tendency to accumulate in post-mitotic cells over time (Brunk and Terman 2002a) and may therefore serve as an aging biomarker (Gray and Woulfe 2005). Theoretically, in highly proliferative cell types, lipofuscin pigments may become diluted as cells divide by mitosis.

### Where and how is lipofuscin formed?

Lipofuscin is present within secondary lysosomes and residual bodies, two stages of respectively mature and aging, ‘retired’ lysosomes. Primary lysosomes are membrane-bound organelles containing active digestive enzymes originating from the trans-Golgi network. They become secondary lysosomes when taking up material upon fusion with phagosomes or endosomes (Terman and Brunk 2005). The lysosomal enzymes have a characteristic acid pH optimum to digest and recycle the ingested material (Terman and Brunk 2005). However, when indigestible material has been taken up, its storage might lead to lysosomal storage diseases such as Gaucher, Fabry, Pompe, and Niemann–Pick (Gragnaniello et al. 2025; Ferreira and Gahl 2017).

Proteins, nucleic acids, carbohydrates, and lipids are molecules digested by lysosomes. Lysosomal digestion also includes autophagy, a digestion of organelles and parts of the cytoplasm contributing to the rejuvenation of cellular organelles. This process becomes less effective with age and disease (Wang and Robbins 2014), but is nevertheless considered to be a contributor to the formation of lipofuscins. Defective mitochondria (Wang and Robbins 2014) or aberrant protein aggregates (Song et al. 2023; Höhn and Grune 2013) are also processed by autophagy. It is generally accepted that lipofuscin comprises a complex mixture of fragmented subcellular protein components, lipids, and other yet unidentified cellular debris resulting from incomplete degradation by secondary lysosomes (Brun and Brunk 1970).

Normally, the acidic and reducing matrix of lysosomes mediates the enzymatic degradation of organelles, proteins, and macromolecules (Brunk and Terman 2002a). However, iron-containing proteins, such as cytochromes and ferritin, release reactive iron which interacts with hydrogen peroxide to produce damaging hydroxyl radicals, a process known as the Fenton reaction (Brunk et al. 1992). The free radicals cross-link amino groups of proteins contributing to the undegradable nature of lipofuscin. Lipofuscin aggregates accumulate within lysosomes, occupying up to 75% of their volume which impairs autophagy (Terman and Brunk 1998a, b). Experiments demonstrate that lipofuscin-loaded fibroblasts had reduced lysosomal proteolytic enzyme activity (Terman et al. 1999; Terman and Brunk 1998a).

Cellular accumulation of free radicals was described in the ‘free-radical theory of aging’ approximately 70 years ago (Harman 1955). This theory postulates that cellular aging is the result of free radical damage caused by oxygen radicals that interact with iron to generate hydroxyl radicals. This subsequently led to the development of the mitochondrial-lysosomal axis theory proposed by Brunk and Terman in 2002, which implicates a double-hit process where oxidative damage of the mitochondria and the inability to remove byproducts contribute to the storage of lipofuscin (Brunk and Terman 2002b). Lipofuscin-loaded lysosomes are proposed to act as an ‘enzyme sink’, diluting functional enzymes from secondary lysosomes and hoarding enzymes within residual bodies. The inability of the enzyme to degrade newly autophagocytosed debris forms a step in promoting oxidation to form more lipofuscins. Loading fibroblasts with lipofuscin reduces their survival by impairing autophagy (Terman et al. 1999).

### How are lipofuscins visualized?

Under the light microscope, lipofuscin is typically observed as a yellow–brown to brown pigment in hematoxylin and eosin (H&E)-stained sections. Specific histochemical protocols have been developed for lipofuscin, and its colored appearance varies depending on the histological staining procedure (Table 1). Lipofuscin is composed of a mixture of oxidized proteins and lipids with lesser contributions from carbohydrates and metals (Porta 1991), such as iron and traces of copper and zinc (Terman et al. 2010). Additionally, lipofuscin contains numerous carboxyl groups, which make it basophilic and allows it to interact with dye cations (Kiernan 2015). As such, lipofuscin can be detected using a series of classical histochemical staining methods, such as Sudan Black, Fontana–Masson, Schmorl, Ziehl–Neelsen, periodic acid–Schiff (PAS), and ferric ferricyanide. Coloration substances are carbohydrates, lipids, and metals (for an overview of available methods, see Table 1). While compiling Table 1, it became clear that lipofuscin varies by cell type and the chosen histochemical method. However, Sudan Black appears to be the stain of choice for specifically demonstrating lipofuscin as a hydrophobic pigment next to the excellent color contrast generated by the dye’s counterstaining properties.

**Table 1:** An overview of histochemical methods for visualizing lipofuscin

Histological stain	Color appearance	Comments	Literature reference
Ferric ferricyanide	Green	Primarily used to detect iron deposits in tissue sections which appear blue. Pigment readily visible for the trained eye because of low contrast	(Adams 1956; Fakan and Chlumská 1997);( Kiernan 2015)
Fontana–Masson	Black	Silver-based stain used to demonstrate melanin, lipofuscin, and other pigments in tissue sections	(Dayan et al. 1988); (Bancroft and Gamble 2008)
Gomori trichrome	Orange–brown	Used in liver fibrosis and muscle degeneration, it highlights various tissue and cytoplasmic components, with pigment visible due to excellent contrast	(Ding et al. 2010)
Hematoxylin (and eosin)	Yellow–brown	Basophilic stain binds acidic components, with pigment easily observed due to contrast from dye counterstaining	(Lillie 1969; Exbrayat 2013)
Lillie’s Nile Blue Sulfate	Blue	Replaced the carbol fuschin in the Ziehl–Neelson stain (vide infra) with Victoria blue	(Lillie 1956)
Luxol Fast Blue	Blue	Has a high affinity for phospholipids. Mainly used in neuropathology to stain myelin	(Exbrayat 2013)
Masson–Fontana	Brown–black	Typical Ag-reduction stain for argentaffin cells and melanin, with incidental observation of lipofuscin	(Dayan et al. 1979)
Nile Blue A	Dark blue	Stains phospholipids, neutral lipids, and lipofuscin, with strong fluorescence emitting in the 600–700 nm range	(Bancroft and Gamble 2008)
OsO_4_	Black or brown	Primarily used as a fixative in TEM, OsO_4_ binds to unsaturated fatty acids, decorating cell membranes and lipofuscin black. In toluidine blue-stained sections, they appear dark brown	(Gouras et al. 2018); Fig. 2
Period acid–Schiff	Magenta–purple	Though not the primary method, it may stain magenta or purple due to lipid oxidation. Combining with other stains is recommended	(Dayan et al. 1988; Kiernan 2015)
Sudan Black B	Blue–Black	Stains hydrophobic cell components (e.g., triglycerides, phospholipids, myelin sheets, lipofuscin) and quenches fluorescence	(Evangelou and Gorgoulis 2017; Dayan et al. 1988; Davan-Wetton and Montero-Melendez 2024)
Schmorl’s ferric-ferricyanide reduction	Yellow–brown	Difficult to distinguish between bile and lipofuscin in hepatocytes. Note: also stains melanin	(Bancroft and Gamble 2008; Eguchi et al. 2021)
Thionine	Green	Though recommended, it is rarely used. Stains other lipid-rich structures pink in frozen sections	(Exbrayat 2013)
Ziehl–Neelson	Magenta	Stain method mainly used in microbiology to demonstrate acid-fast mycobacteria	(Dayan et al. 1988; Exbrayat 2013; Goldfischer and Bernstein 1969)

Alphabetically listed histochemical stains to aid lipofuscin visualization. Note that some of the stains also allow lipofuscin to be concurrently visualized via its (auto)fluorescence properties. For detailed protocols on histochemical and related lipofuscin-staining methods, see Bancroft and Gamble 2008, Exbrayat 2013, and Kiernan 2015. For the interested reader, the ‘Special Lecture’ paper by Ryuei Maeda (Maeda 1965) describes in detail the historical identification of histochemical stains of ceroid and lipofuscin. In this paper, a detailed comparison is provided regarding histochemical differentiation among different species, organs, and the use of various dyes

Lipofuscin also has fluorescent properties with an excitation wavelength between 320 and 480 nm and emission wavelength between 460 and 630 nm (Brunk and Terman 2002a; Csallany and Ayaz 1976; Yin and Brunk 1998). Lipofuscin’s auto-fluorescence is attributed to fluorescent Schiff bases from protein and lipid oxidation induced by free radicals (Chio et al. 1969; Chelh et al. 2007). As a result, lipofuscin is often observed in a variety of tissues as an autofluorescent pigment that overlaps with the emission range of commonly used secondary antibodies. To avoid misinterpretation during antibody labelling of intracellular structures, numerous studies have aimed to reduce lipofuscin autofluorescence using copper sulfate, Sudan Black, ammonium acetate, ammonia–ethanol, and sodium borohydride (Schnell et al. 1999; Baschong et al. 2001; Oliveira et al. 2010). Sudan Black was the most effective at eliminating autofluorescent signals in the brain and myocardium (Oliveira et al. 2010; Baschong et al. 2001), as well as liver, kidney, and pancreas when combined with UV radiation (Viegas et al. 2007). Sodium borohydride is a strong reducing agent and is often used in immunolabeling workflows to block free aldehyde groups and increase specific labelling in formaldehyde- or glutaraldehyde-fixed tissue (Baschong et al. 2001). Unfortunately, the compounds listed above also reduced the overall intensity of the fluorophores (Schnell et al. 1999).

Besides the challenges posed by the autofluorescent properties of lipofuscin, antibodies against this aging pigment are largely absent as a result of tissue-specific variations of their exact biochemical composition (Sjöstedt 2024). Since 2016, several antibodies were generated by the Human Protein Atlas targeting components of lipofuscin including antibodies against HDGFL1, FAM96B, and GGH (Evangelou and Gorgoulis 2017; Sjöstedt 2024). Not only have these antibodies not been extensively characterized but an independent group has suggested that autofluorescence properties may provide a more accurate reflection of lipofuscin concentration (Bertolo et al. 2019). In daily practice, histochemical stains and autofluorescence remain the common approach to examine lipofuscin in tissue and cells (Jung et al. 2010). Although readily visible with the light microscope, transmission electron microscopy (TEM) conclusively identifies their complex fine structural appearance (vide infra).

Lipofuscin quantification is based on a relative scale, which makes comparisons between species and organs challenging. Different levels of accumulation were noted under both autofluorescence and histological stains comparing the same organs in humans and rats (Monserrat et al. 1995). Variability between histological stains in the same cell type are also not unexpected given their variable composition. For example, notable variations in saccharides were identified in lipofuscin from different cell types, but also from the same cell type of different species (Porta 2002). Similarly, mannose is one of the most consistently detected sugars present in lipofuscin in almost all cell types except for choline-deficient hepatic lipofuscin in rats (Monserrat et al. 1995). In hemochromatosis, iron deposits also stain brown in cardiomyocytes. This could easily be mistaken for lipofuscin under H&E staining. To resolve this problem, further development of histochemical stains for iron is necessary (Fishbein et al. 2024).

TEM offers an effective alternative visualization method for imaging lipofuscin. The earliest TEM studies appeared in the 1960s (e.g., Essner and Novikoff 1960; Björkerud 1963; Malkoff and Strehler 1963; Abrahams et al. 1964). They described lipofuscin as a heterogeneous electron-dense pigment within different cell types. From that moment onwards, many TEM papers have been published on lipofuscin in the heart, liver, brain, retina, and skin. Depending on the cell type, these pigments vary in size (0.5–5 µm in diameter). Owing to its contrasting interaction with osmium tetroxide and lead citrate and uranyl acetate, TEM also contributed to the understanding of the unsaturated (phospho)lipid and protein content of lipofuscin. Essner and Novikoff (1960) combined cytochemical techniques with TEM and found that the pigmented areas in the liver also showed high acid phosphatase and cathepsin activity. This observation confirmed the important relationship between lipofuscin and lysosomes.

Evidently, the power of microscopic visualization lies in the ability to combine microscopy and histochemistry to evaluate clinical tissues (Shami et al. 2017; Keuenhof et al. 2021). One good example is the case report by Leung et al. (Leung et al. 2019) in which the authors applied six different histochemical staining protocols, immunofluorescence labelling, and TEM. They studied the distribution of lipofuscin and other cellular inclusions over time in kidney biopsies from a patient who received a donor kidney. This study clearly demonstrated a relationship between inflammation, oxidative stress, and lipofuscin within the renal tissue.

A noninvasive imaging approach has recently been described that utilizes label-free imaging of lipofuscin at near- and shortwave-infrared wavelengths to quantitatively assess the degree of pigment accumulation in fibrotic vs. cirrhotic livers (Saif et al. 2020). The authors showed great promise for this imaging approach in vivo on mice and on human biopsies in situ.

### Can lipofuscin be studied outside of tissue?

The cellular accumulation of lipofuscin is a slow and protracted process. Although methods to purify lipofuscin from tissue or to artificially induce lipofuscin in vitro were developed, Siakotos and colleagues were, to the best of our knowledge, the first to describe a method for isolating and purifying the pigment based on enzymatic digestion of tissues (Siakotos et al. 1973; Siakotos and Strehler 1974). This study was conducted on healthy humans’ heart and liver tissue with no history of disease and was obtained through autopsy. TEM confirmed the isolated fractions were pure and comparable in structure to lipofuscin within cells.

Nilsson and Yin (1997) were able to induce lipofuscin production by exposing liver nuclei, mitochondria, lysosomes, and microsomes, isolated from homogenized rat liver slices by sucrose-gradient fractionation, to UV light. TEM investigation confirmed the characteristic lamellar structures and osmiophilic lipofuscin nature of the preparations. Elemental X-ray analysis showed that the material contained calcium and iron, similar to naturally occurring lipofuscin. Furthermore, the material displayed autofluorescence with a fluorescence maximum at 430 nm when excited at 350 nm which is similar to the autofluorescence properties of lipofuscin found in tissues. The authors showed that cultured human fibroblasts endocytosed the material, resulting in lipofuscin-loaded, aged-like cells. Subsequent studies confirmed that high-grade lipofuscin could be generated from purified mitochondria from mouse (Gray and Woulfe 2005) and rat liver (Frolova et al. 2015). Certain contents within isolated mitochondria can be cross-linked under UV irradiation creating a stable polymer, as can also be done with the application of heat which oxidizes lipids (Frolova et al. 2015).

Lipofuscin can also be experimentally induced and manipulated in cell cultures (i.e., in vitro) by oxidative stress (Brunk and Terman 2002a). Oxidative stress models typically employ hyperoxia to enhance free radical formation in cultured cells which may be perpetuated by UV irradiation to generate artificial lipofuscins (Yin and Brunk 1998; Sohal et al. 1989). In neonatal rat cardiomyocytes, for example, lipofuscin begins to form after 2 weeks in hyperoxia culture with 40% oxygen, but the levels do not stabilize until 4 weeks in culture (Brunk and Terman 2002a). If lipofuscin was purely the result of oxidative stress, then the addition of chelators, which function as oxygen scavengers, should rescue the cells. In vitro lipofuscin models revealed that some, but not all, chelators could effectively reduce lipofuscin accumulation. For example, desferrioxamine and DTPA had pronounced effects at high oxygen saturations, whereas EDTA did not (Marzabadi et al. 1990). To accelerate lipofuscin formation, Fe, Al, Cd, Hg, or Pb metals can be added to the culture medium, combined with a high oxygen level (Marzabadi and Jones 1992; Marzabadi et al. 1992). More recent studies induced cellular senescence using chemical agents such as paraquat (Baldensperger et al. 2024; Höhn et al. 2010, 2012; König et al. 2017) or glucose starvation coupled with doxorubicin (Song et al. 2023) all promoting lipofuscin formation.

## Lipofuscin in liver biology

**Fig. 2:**
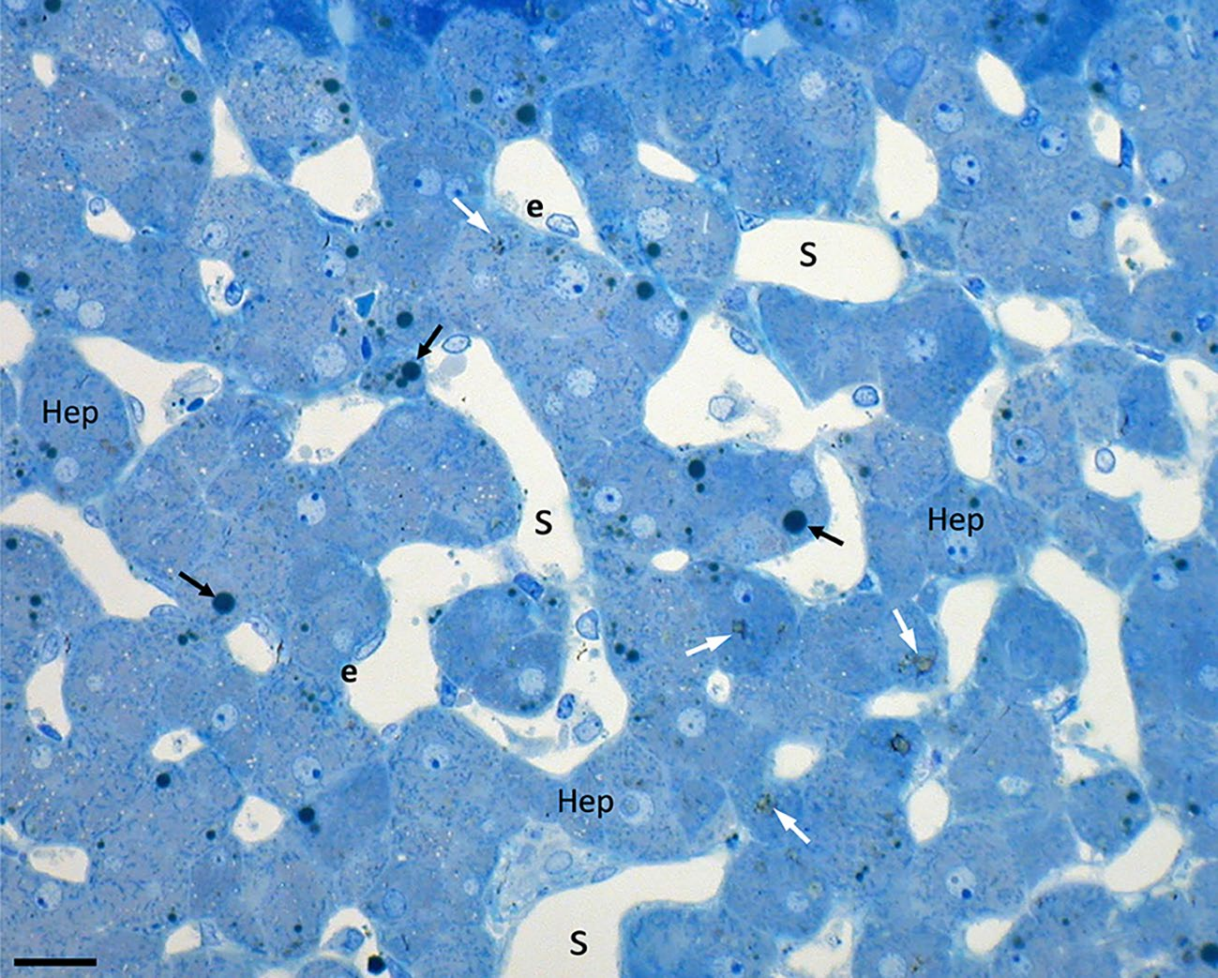
Wide-field light optical image of a semi-thin section of a glutaraldehyde and osmium tetroxide fixed human liver tissue stained with toluidine blue. This liver tissue was confirmed to be positive for lipofuscin, as determined by electron microscopy (see Fig. 3 for comparison). White arrows denote examples of lipofuscin inclusions within the cytoplasm of hepatocytes; black arrows point to lipid droplets. Note that the lipofuscin inclusions appear in various irregular shapes, containing both darker lipid material and lighter-colored matter. In contrast, lipid droplets display a more homogeneous content, rounded shape, and staining pattern. *Legend*: e, liver sinusoidal endothelium; Hep, hepatocytes; S, liver sinusoids. Scale bar, 25 μm

**Fig. 3:**
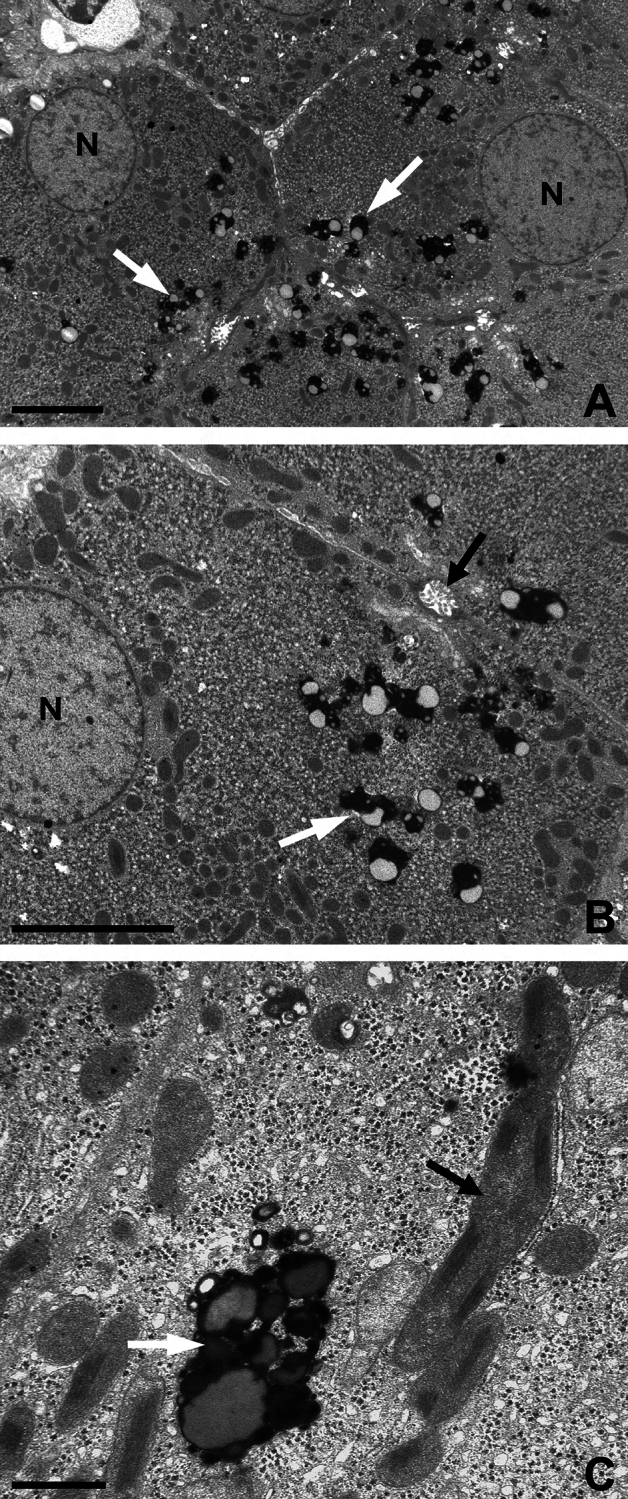
Transmission electron microscopy images of lipofuscin inclusions observed within liver sections of patients with metabolic-associated fatty liver disease. **A** Low-magnification overview image showing an abundance of lipofuscin inclusions within the liver parenchymal cell cytoplasm (white arrows). Their characteristic morphological appearance in transmission electron microscopy indicates that they are composed of a type of lipid droplet (i.e., exhibiting an electron-dense appearance) along with material of intermediate electron density. Lipofuscin inclusions are also referred to as residual lipofuscin bodies or lipofuscin pigments, which are essentially senescent lysosomes containing residual accumulations of cellular matter. Scale bar, 5 μm. **B** Intermediate-magnification image of two neighboring liver parenchymal cells. Note the accumulation of lipofuscin (white arrow) around a bile canaliculus (black arrow). Their presence around bile canaliculi is frequently observed. Scale bar, 5 μm. **C** High-power magnification of lipofuscin bodies (white arrow). A second frequent occurrence presents itself and involves the adjacent presence of giant mitochondria (black arrow) in the vicinity of lipofuscin inclusions. Note the remarkably elongated and well-organized crystal-like inclusions within the mitochondrial matrix. Samples were prepared as detailed in the recent work by Verhaegh et al. (2021) and Wisse et al. (2022). *Legend*: N, nucleus. Scale bar, 1 μm

To the best of our knowledge, the first description of ceroids in liver tissue was given by Lillie et al. using rats suffering from alcohol-induced cirrhosis. Lillie was also recognized as the namesake behind the Lillie blue stain (Table 1), which was used to visualize these wax-like pigments, later determined to be lipofuscin (Lillie 1956; Lillie et al. 1942, 1941). Lipofuscin has a characteristic appearance in human parenchymal cells. Light microscopy reveals lipofuscin as a dark wear-and-tear pigment, depending on the histochemical staining protocol used (Table 1 and Fig. 2). TEM provides a much higher detail of its granular, heterogeneous, electron-dense contents (Fig. 3). Across various reports, lipofuscin consistently reveals lipid-rich matter contained within residual bodies (retired lysosomes).

### The aging liver

One of the early papers on liver lipofuscin is by Bachmann (1953), in which he acknowledges lipofuscin as a hallmark of liver adversity. He based his conclusions on the histochemical investigation of 712 liver biopsies from 562 young to old patients. However, repeated biopsies revealed that, irrespective of age, the pigment can decrease relatively quickly but can also reappear rapidly. This suggests that the pigment is more a result of organ stress rather than an indicator of aging.

Lipofuscin accumulation was found across rat liver, heart, kidney, and brain tissues of different ages (2, 11, 30 months) (Ikeda et al. 1985). Three types of lipofuscins were described by TEM: type 1, early-phase electron-dense granular bodies; type 2, homogenous structures surrounded by indistinct limiting membrane; and type 3, near-mature lamellated with cristae-like mitochondria (which we now recognize as autophagic vacuoles) and combinations of the aforementioned structures. The highest accumulation of lipofuscin was present in Purkinje cells and kidney cells with predominant type 1 inclusions that increased with age. Liver and heart had similar abundances of combined type 1 and type 2 inclusions. In liver parenchymal cells, lipofuscin was observed most frequently in the pericanalicular region where they were surrounded by lamellae of the Golgi apparatus and mitochondria (Ikeda et al. 1985). In a follow-up study, the research team of Ikeda demonstrated a clear association between lipofuscin and food intake during calorie-restriction experiments in aging rats (Iwasaki et al. 1988). Lipofuscin granules as observed in the TEM were increased in animals with unlimited access to food as they aged. This is one of the first papers to document that food restriction delays certain age-related changes in liver parenchymal cells.

A biochemical study confirmed that lipofuscin in liver homogenates increased with age when comparing young (2–8 months) and older (12–24 months) rats (Szweda 1994). Another observation from this study is the autofluorescence of lipofuscin and that of retinyl palmitate in hepatic stellate cells are due to the presence of vitamin A. The autofluorescence of liver homogenates may therefore also be attributable to vitamin A instead of lipofuscin.

In human gallstone disease, accumulations of lipofuscin were observed in the centrilobular areas of 196 liver biopsies of patients between 22 to 92 years of age. Nevertheless, these livers were considered histologically normal (Brændstrup and Dombernovsky 1970). Amongst the 196 liver samples, 80 revealed undiagnosed portal fibrosis and fatty infiltration or cholestasis. Age was a significant predictor of lipofuscin deposition. The number of biopsies with high levels of lipofuscin jumped from 13% in the 20-year-old group to 80% in the above 70-year-old group. An interesting note is the fact that women were predisposed to higher levels of lipofuscin. Furthermore, lipofuscin accumulation was much more prominent in samples with normal as compared to abnormal liver tests as measured by serum alkaline phosphatase and icterus index. This and other groups suggest a counterintuitive idea where liver pathologies disrupt lipofuscin accumulation (Bachmann 1953; Tygstrup et al. 1965; Brændstrup and Dombernovsky 1970).

Tauchi et al. (1980) investigated the genetic and environmental influence of lipofuscin accumulation. The authors studied the distribution of lipofuscin in the central vs. peripheral zones of more than 300 livers of American Caucasians, Hawaii Japanese, and native Japanese populations. Pigment was age-related in some cases, but was influenced by the nutritional condition, the constitution of the individual, and the population to a greater extent. Lipofuscin was the lowest in Japanese, followed by American Caucasians. Therefore lipofuscin was not only part of aging but also of geographical or racial circumstances (Tauchi et al. 1980).

Yokota (1980) utilized light microscopy to provide an area/density scoring of lipofuscin in human liver, heart, and adrenal gland between the ages of 0 and 84 years. Lipofuscin was positively correlated with aging. The highest levels were found in the heart, followed by liver and then adrenal glands at a 3:2:1 ratio. When lesions or diseases were present, lipofuscin was lower than in the normal liver. Lipofuscin was more abundant in the centrilobular regions of the liver than peripheral regions. With the exception of diseased liver, no relationship with disease or environmental factors (even in atomic bomb survivors) was found in any organ.

Recently, Chatterjee et al. (2024) consider the human liver as a ‘youthful organ’, with hepatocytes averaging only a few years of age. The authors review in detail the ‘subtle changes’ that occur with increasing age, such as blood flow, detoxification, pseudo-capillaries, and excess lipid deposition and lipofuscin. The authors present insights into the processes that allow liver tissue to regenerate in response to adversity. They support the idea that lipofuscin is an indicator of cellular stress and aging of cells, although the presence of lipofuscin may be temporary in nature as with hepatic parenchymal cells in children with cystic fibrosis (Hultcrantz et al. 1986).

From the literature cited above, the presence of lipofuscin correlates with cellular functionality and longevity. It is well known that organs, tissues, and cells age at different rates. Liver parenchymal cells, long valued for their regenerative capacity, may dilute lipofuscin levels through cell multiplication, potentially reducing its role in cellular damage. Almost half of a healthy human liver can be transplanted with the capacity to grow back to its original size within 3 months, this regeneration is even faster in rats: it lasts weeks instead of months. As a result of this enormous regenerative capacity, lipofuscin is not expected to accumulate in the liver until such time that chronic disease impairs their proliferative capacity.

### The diseased liver

The rate of lipofuscin accumulation with age in liver is lower than other organs, which is probably caused by cell division. Nevertheless, lipofuscin can be seen in pathological conditions such as chronic liver disease (e.g., cirrhosis, hepatitis), metabolic disorders (e.g., steatosis, alcoholic liver disease), genetic disorders (e.g., Wilson’s disease, hemochromatosis), and drug- and toxin-induced injury.

The study by Essner and Novikoff (1960) suggests a transition from ‘dense bodies’ to lipofuscin pigment. These lipofuscins containing electron-dense bodies are mainly observed around bile canaliculi. It is known that bile canaliculi drain the liver from substances accumulated in peribiliary lysosomes. Their fine structure is typically characterized by ‘multilobulated’ inclusions. Enzyme cytochemical studies on patients with idiopathic jaundice indicated that lipofuscin inclusions originate from lysosomes. At that time (1960), the authors raised the question to gerontologists whether lipofuscin could also be found in heart cells.

These observations were supported by the paper of Dubin and Johnson (1954) which examined 12 young patients with idiopathic jaundice. The healthy-looking liver of these patients did not show obstruction or inflammation but had elevated levels of bilirubin and granular brown pigment in liver parenchymal cells (Dubin and Johnson 1954). The unidentified brown pigment shared qualities with ceroids and cardiac lipofuscin with positive PAS staining indicating oxidation of unsaturated fatty acids. The authors suggested the name ‘mesobilifuscin’ for the brown pigment and that it could be derived from the breakdown of hemoglobin (Dubin and Johnson 1954).

Similar observations were performed in 26- to 43-week-old neonatal human liver in hepatocytes and bile duct cells around the hepatic triad (Goldfischer and Bernstein 1969). Intriguingly, the level of copper in the inclusions mentioned was approximately ten times higher than in adults. Whether metals contribute to the generation of lipofuscin remains unknown. Lipofuscin was mentioned to be autofluorescent but in some instances this fluorescence can be quenched by copper and iron (Barka et al. 1964). Goldfisher and Bernstein even argued that lipofuscin should not be called the ‘senescent wear and tear pigment’, because it was regularly found in newborns, infants, and adolescents.

Satodate and Terui analyzed 65 human autopsy livers with different diseases (Satodate and Terui 1965). In this rigorous investigation a number of livers contained the pigment spanning different diseases and ages (3 months–76 years). At the time, the term ‘ceroid’ was still commonly used to describe the ‘yellow aging pigment’. The researchers found the pigment in more than a quarter of the livers with biliary cirrhosis and cirrhotic livers in infancy and childhood. Using a panel of stains, the researchers detected pigment in two cell types: liver parenchymal cells and Kupffer cells (resident liver macrophages).

In patients suffering from primary biliary cirrhosis, TEM-based X-ray analysis demonstrated the presence of Ca, P, K, Cl, S, Al, and Cu in lipofuscin inclusions (Humbert et al. 1982). The authors postulated that the presence of sulfur suggests the occurrence of metalloproteins binding these elements. Hepatocyte metalloproteins play a key role in detoxification, metabolism, and oxidative stress. Metalloproteins are also important scavengers for reactive oxygen species that play a role in lipofuscin formation. So, it was concluded that copper protects the liver from adversity. A significant amount of copper has also been observed within hepatocellular lipofuscin by X-ray TEM analysis of a 17-year-old male patient suffering from Wilson’s disease (Motonishi et al. 2006).

In patients with α1-antitrypsin deficiency and minimal liver disease, TEM revealed increased lipofuscin in the lysosomes of liver parenchymal cells, regardless of whether the patients were homozygous or heterozygous for α1-antitrypsin deficiency (Hultcrantz and Mengarelli 1984). Another example of a genetic disorder is primary hyperoxaluria type I, in which all patients showed conspicuous amounts of lipofuscin. The authors ascribed this to the various metabolic disturbances to which the livers were subjected during the course of the disease (Iancu and Danpure 1987).

High levels of hepatocellular lipofuscin were observed in patients with nephritis upon chronic and excessive analgesic intake (Abrahams et al. 1964). TEM data of liver parenchymal cells showed a high degree of swelling of the endoplasmic reticulum. This caused compression of the mitochondria, including degeneration of the mitochondrial membranes and their cristae. The authors noted an abundance of lipofuscin throughout the cytoplasm of the liver parenchymal cells. When compared to healthy patients, drug-induced liver toxicity was proposed as the causal source of lipofuscin formation.

The presence of lipofuscin has also been demonstrated in the liver of zebrafish (*Danio rerio*) (Gandahi et al. 2020). Lipofuscin was observed in the cells that make up the melanomacrophagic centers (MMCs). Mitochondrial degeneration was observed accompanied by lysosomal digestion in MMCs, together with structural changes of the rough endoplasmic reticulum. The authors concluded that liver-resident MMCs contribute to the clearance of aged organelles, and that lysosomal digestion eventually results in the presence of lipofuscin. This is consistent with the observation in humans and experimental animals. Similar studies on zebrafish found an increasing amount of age-related lipofuscin in liver parenchymal cells (Kishi et al. 2009) and skeletal muscle tissue (Kishi et al. 2008) which is relevant considering the lifespan of zebrafish (3.5–5 years). In early larval stages of zebrafish (1–12 days post-fertilization), lipofuscin granules are absent in the liver, as observed by fluorescent and electron microscopy (Cheng et al. 2017, 2019, 2020).

In a contribution by Winter (*Nature* 1961) lipofuscin pigmentation of sheep livers was related to their food intake (Winter 1961). Similar to other studies (Satodate and Terui 1965), 18 stains were employed to detect yellow–brown, 2-micron lipofuscin granules in various stages of oxidation. The pigment was clearly present in sheep from the far northeast coast of Australia. This suggested that the sheep had been grazing on mulga wattle (*Acacia aneura*) for extended periods of time before slaughter. This situation was absent in other abattoirs in Australia, where sheep were slaughtered immediately. It can also be suggested that a food ingredient caused cellular stress, leading to the liver discoloration. In a similar study, ‘hepatic lipofuscinosis’ was described in liver parenchymal cells and Kupffer cells of healthy Norwegian sheep (Nordstoga 1990).

The book chapter titled ‘Electron microscopy of liver biopsies’ by Iancu and Manov (2011) provides TEM illustrations that describe the ultrastructure of lipofuscin in a number of liver diseases. TEM illustrations include Reye syndrome, metabolic-associated fatty liver disease, Dubin–Johnson syndrome, drug toxicity, parenteral nutrition, and ceroid lipofuscinosis. Notably, lipofuscin inclusions are often associated with large lipid droplets with different electron density. The paper by Iancu et al. (2013), describing the ‘lipolysosome’ in fatty livers of patients of varying ages, clearly demonstrates a structural relationship between the pigment and large fat deposits in liver parenchymal cells.

**Fig. 4:**
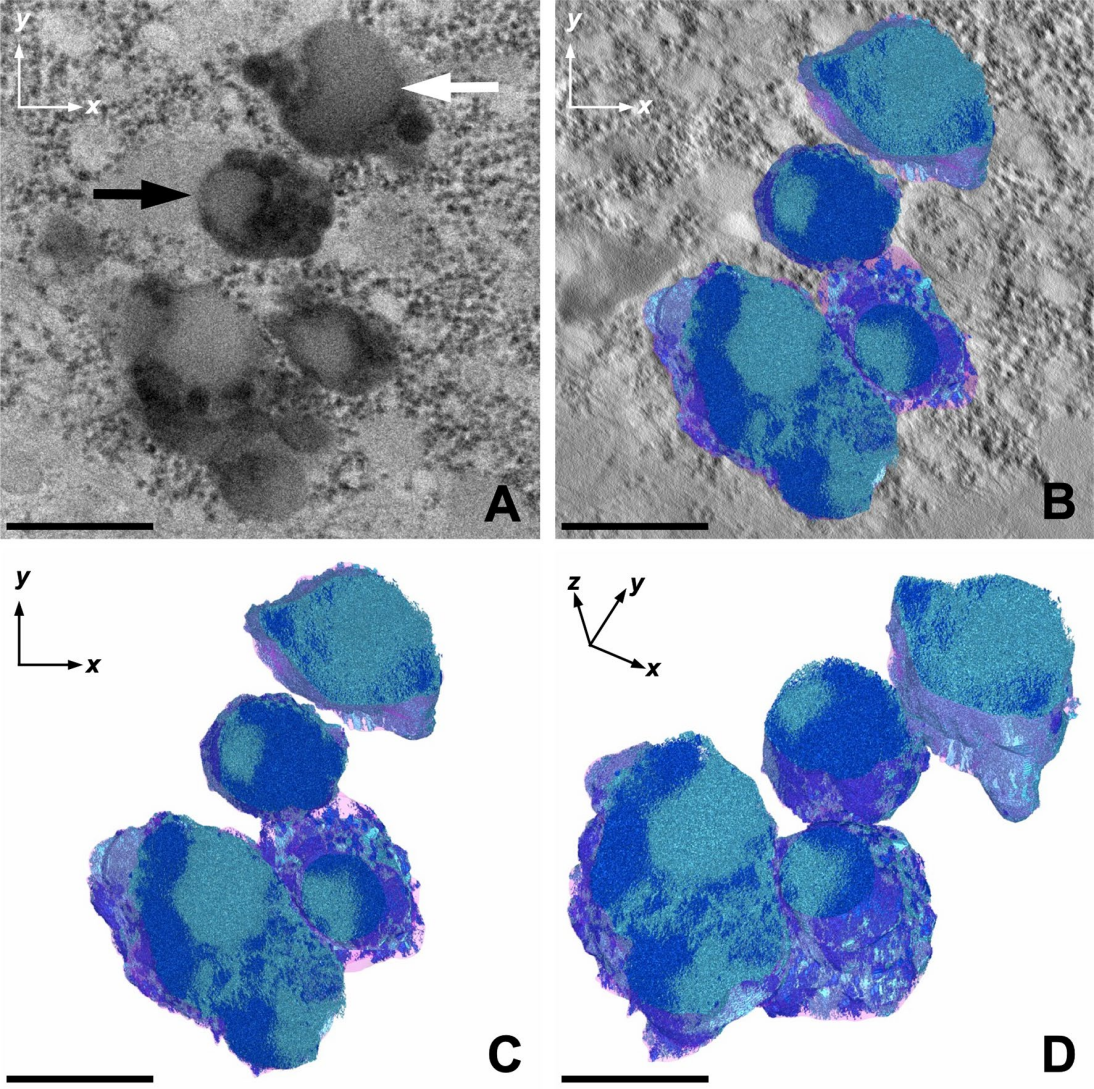
Transmission electron microscopy (**A**) serial-section electron tomography (**B**–**D**) of representative lipofuscin granules in human liver tissue (see also, Supplementary Information — Video). **A** High-magnification transmission electron micrograph showing marked variation in electron density within lipofuscin granules, ranging from electron-lucent to electron-dense granular material, consistent with the heterogeneous composition of the ‘residual waste body’. The white arrow indicates a granule (referred to as granule 1 in the manuscript text) predominantly composed of electron-lucent material (78.6%; 0.2431 µm^3^), whereas the black arrow denotes a granule (designated as granule 2 in the manuscript text) predominantly composed of electron-dense material (86.8%; 0.0911 µm^3^). **B** Rendered 3-D model overlaid on the 2-D micrograph shown in **A**. **C**, **D** 3-D models of panels **A** and **B**, from which image **D** is depicted under a different angle, illustrating the fine structural organization and content of lipofuscin granules. The 3-D model highlights the spatial distribution of electron-lucent (light blue) and electron-dense (dark blue) matter that make up the lipofuscin granules. The magenta color represents the limiting membrane. Scale bars, 1 µm

Finally, we applied serial-section electron tomography, one of the available volume electron microscopy approaches that has proven to be a powerful imaging method for revealing subcellular three-dimensional (3-D) information at the nanometer scale within its structural context (Eisenstein 2023). More specifically, serial-section TEM was applied to samples as shown in Fig. 3. To the best of our knowledge, Fig. 4 provides the first detailed glimpse of lipofuscin granules within the 3-D architectural context of these membrane-bound inclusions in resin-embedded human liver biopsy tissue (see also, Supplementary Information — Video). This is, to some extent, unsurprising, given that these structures—often informally described as the cell’s “garbage bins”—have historically received limited scientific attention. In this example, volumetric analysis of the sampled regions revealed that the electron-dense osmiophilic components accounted for 50.3% (0.3451 µm^3^), while electron-lucent components comprised 49.7% (0.3409 µm^3^). At the level of individual granules, however, composition varied markedly. More specifically, comparison of two randomly selected granules highlights a striking contrast (see Fig. 4A for details on granule 1 vs. granule 2), with granule 1 being predominantly electron-lucent (78.6%; 0.2431 µm^3^) and granule 2 predominantly electron-dense (86.8%; 0.0911 µm^3^). These values should be interpreted in the context of partial sampling of each granule, and a more rigorous assessment of lipofuscin ultrastructure will require analysis across a larger cohort of structures in future studies and under different experimental or clinically relevant conditions. Irrespective, future volume TEM studies would certainly contribute to a better understanding of the formation of lipofuscin, especially when combined with elemental mapping to examine the fine 3-D chemical composition of the pigment and other closely associated ingested matter within these residual waste bodies (Scotuzzi et al. 2017). These new structure–function insights—particularly when combined with omics approaches (Son et al. 2024) and considered alongside variations in patient antecedents—may contribute to the development of novel treatment regimens aimed at arresting or even reversing senescence or aging.

## Conclusion

Herein, we highlighted the structure and function of lipofuscin in the liver (summarized in Table 2), which seems to be more significantly impacted by disease than aging. While lipofuscin is commonly regarded as a marker of cellular aging, its specific impact on organ function remains relatively poorly understood, given the limited research available. From this review, it became clear that lipofuscin accumulation may correlate with oxidative stress, potentially contributing to long-term liver dysfunction. However, the precise mechanism by which lipofuscin affects liver disease progression remains unclear. Further research is needed to delineate the pathways through which lipofuscin affects cellular function and to explore its potential as a drug target—either by directly modulating lipofuscin or by targeting the associated intracellular pathways and organelles. For example, this would be highly beneficial in the context of metabolic dysfunction-associated steatotic liver disease (MASLD)—formerly known as non-alcoholic fatty liver disease (NAFLD)—and the presence of lipofuscin, together with a better understanding of it, might lead to improved insights into the development of new diagnostic or therapeutic approaches specific to MASLD. Supporting this, a promising study by Li et al. (2021) showed that long-term administration of the off-label longevity drug rapamycin reduces lipofuscin accumulation in the aging heart by enhancing autophagy. Prevention of lipofuscin accumulation may be more effective than removing already accumulated lipofuscin given its undegradable nature. This way might open an interesting avenue for drug discovery research focused on preventing lipofuscin buildup in the diseased liver.

**Table 2:** Overview of key literature on lipofuscin in liver parenchymal cells

Species/model	Disorder & essential finding	Imaging/technique	Reference
Rat	Cirrhosis. First description of ‘ceroids’ in the liver	LM	(Lillie et al. 1942)
Human	Age. Describes the presence of pigment in both young and elderly individuals. It also puts forward the cell stress hypothesis	LM	(Bachmann 1953)
Human	Idiopathic jaundice and hyperbilirubinemia. Hypothesis that the liver pigment is mesobilifuscin originated from hemoglobulin	LM	(Dubin and Johnson 1954)
Human	Obstructive and chronic idiopathic jaundice. First description that lipofuscin is associated with lysosomes through cytochemistry	TEM	(Essner and Novikoff 1960)
Sheep	Food. Grazing certain vegetation resulted in hepatocellular stress and occurrence of lipofuscin	LM	(Winter 1961)
Human	Nephritis. Analgesic-induced kidney damage resulted in the increased presence of lipofuscin within liver parenchymal cells	TEM	(Abrahams et al. 1964)
Human	Cirrhotic (biliary) livers. Lipofuscin is present in this condition irrespective of age	LM	(Satodate and Terui 1965)
Human	Newborn. Demonstrates the ‘wear-and-tear’ pigment in neonates. Lipofuscin is not a measure of cellular senescence	LM & TEM	(Goldfischer and Bernstein 1969)
Human	Gallstones surgery and age. Patients over 60 years show a significant higher level of lipofuscin	LM	(Brændstrup and Dombernovsky 1970)
Human	Age correlates with specific hepatic lesions. Centrilobular area contains more lipofuscin than other regions	LM	(Yokota 1980)
Human	Age, diet, and geographic distribution affect lipofuscin accumulation	FM	(Tauchi et al. 1980)
Human	Primary biliary cirrhosis. Coexistence of minerals, especially sulfur, within lipofuscin inclusions suggests a link with metalloproteins	TEM & XR	(Humbert et al. 1982)
Human	α1-antitrypsin deficiency and minimal liver disease. Significant increased lipofuscin in both periportal and centrilobular regions	TEM	(Hultcrantz and Mengarelli 1984)
Rat	Age. Describes the occurrence of 3 distinct types of lipofuscins based on ultrastructure	TEM	(Ikeda et al. 1985)
Human	Cystic fibrosis. Lipofuscin increased in the pericanalicular region of liver parenchymal cells	TEM	(Hultcrantz et al. 1986)
Rat	Diet and calorie restriction. Unlimited access to food as they age increases amount of lipofuscin	TEM	(Iwasaki et al. 1988)
Rat	Age. Concentration increases with age. Retinyl palmitate, present in vitamin A, has autofluorescent properties	FS	(Szweda 1994)
Human	Wilson disease. High concentrations of iron and copper associated with lipofuscin	TEM & XR	(Motonishi et al. 2006)
Human	Reye syndrome; MAFLD. Presence of a significant amount of lipofuscin	TEM	(Iancu and Manov 2011)
Fish	Age. Adult fish liver contains a significant amount of lipofuscin in melanomacrophagic centers	TEM	(Gandahi et al. 2020)

The above table succinctly summarizes, in chronological order, hallmark ultrastructural and/or biochemical observations of lipofuscin (also known as aging pigment or ceroid) in hepatocytes and cardiomyocytes

*FM* fluorescence microscopy, *FS* fluorescence spectroscopy, *LM* light microscopy, *MAFLD* metabolic-associated fatty liver disease, *TEM* transmission electron microscopy, *XR* X-ray elemental analysis

## Supplementary Information

Below is the link to the electronic supplementary material.

Supplementary file1 (DOCX 756 KB)

Supplementary file2 (AVI 256607 KB)

## Data Availability

F.B., E.W., and A.L. were equally responsible for the acquisition of experimental data presented in Figs. 2 and 3, and G.J.S. was responsible for the data in Fig. 4.
