# Supplementary material for: From lipofuscin accumulation to cellular dysfunction: a focus on liver pathophysiology
Source: Histochem Cell Biol. 2026 Jun 25;164(1):52. doi: 10.1007/s00418-026-02502-9 (PMC13303679; doi:10.1007/s00418-026-02502-9)
Supplement: Supplementary file 1 — Supplementary file1 (DOCX 756 KB) [file 418_2026_2502_MOESM1_ESM.docx]

**Supplementary Information**

**“From Lipofuscin Accumulation to Cellular Dysfunction –**

**A Focus on Liver Pathophysiology”**

**in Histochemistry and Cell Biology**

Filip Braet^1,2^*, Eddie Wisse^4^, Ger H. Koek^4,5^, Gerald J. Shami^1,2^, Amy Li^3,6,7^*

*^1^School of Medical Sciences (Molecular Biomedicine), The University of Sydney, NSW Australia; ^2^Australian Centre for Microscopy & Microanalysis, The University of Sydney, NSW Australia; ^3^School of Medical Sciences, The University of Sydney, NSW Australia; ^4^Division of Nanoscopy, Multimodal Molecular Imaging Institute, University of Maastricht, Maastricht, The Netherlands; ^5^School of Nutrition and Translational Research in Metabolism, Maastricht University, Maastricht, The Netherlands; ^6^Health Hub, Torrens University Australia, NSW Australia; ^7^Department of Rural Clinical Sciences, La Trobe Rural Health School, La Trobe University, Victoria, Australia*

***Corresponding authors**: Filip Braet [filip.braet@sydney.edu.au](mailto:filip.braet@sydney.edu.au) | <https://orcid.org/0000-0002-5222-0895> & Amy Li [amy.li@torrens.edu.au](mailto:amy.li@torrens.edu.au) | <https://orcid.org/0000-0001-5413-3771>


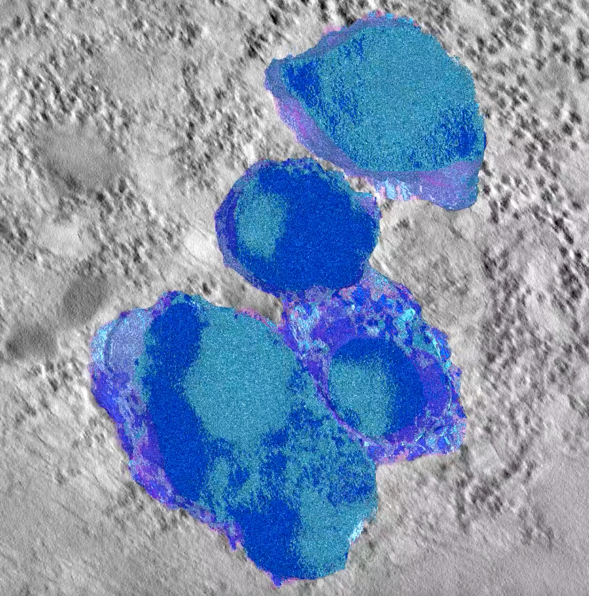


***Supplementary Video***. Animated volume-segmentation data extending the transmission electron microscopy and tomography data presented in Figure 4. Note the variable electron density within the lipofuscin granules, with electron-dense material (dark blue) predominantly localized at the periphery and electron-lucent material (light blue) at the core.
